# Genomic Identification of CCCH-Type Zinc Finger Protein Genes Reveals the Role of *HuTZF3* in Tolerance of Heat and Salt Stress of Pitaya (*Hylocereus polyrhizus*)

**DOI:** 10.3390/ijms24076359

**Published:** 2023-03-28

**Authors:** Weijuan Xu, Shuguang Jian, Jianyi Li, Yusang Wang, Mingyong Zhang, Kuaifei Xia

**Affiliations:** 1 Key Laboratory of South China Agricultural Plant Molecular Analysis and Genetic Improvement & Guangdong Provincial Key Laboratory of Applied Botany, South China Botanical Garden, Chinese Academy of Sciences, Guangzhou 510650, China; 2 University of Chinese Academy of Sciences, Beijing 100049, China; 3 South China National Botanical Garden, Guangzhou 510650, China; 4 CAS Engineering Laboratory for Vegetation Ecosystem Restoration on Islands and Coastal Zones, South China Botanical Garden, Chinese Academy of Sciences, Guangzhou 510650, China; 5 College of Traditional Chinese Medicine, Guangdong Pharmaceutical University, Guangzhou 510006, China

**Keywords:** pitaya, zinc finger protein, salt, heat, *HuTZF3*, *Arabidopsis*

## Abstract

Pitaya (*Hylocereus polyrhizus*) is cultivated in a broad ecological range, due to its tolerance to drought, heat, and poor soil. The zinc finger proteins regulate gene expression at the transcriptional and post-transcriptional levels, by interacting with DNA, RNA, and proteins, to play roles in plant growth and development, and stress response. Here, a total of 81 CCCH-type zinc finger protein genes were identified from the pitaya genome. Transcriptomic analysis showed that nine of them, including *HuTZF3*, responded to both salt and heat stress. RT-qPCR results showed that *HuTZF3* is expressed in all tested organs of pitaya, with a high level in the roots and stems, and confirmed that expression of *HuTZF3* is induced by salt and heat stress. Subcellular localization showed that HuTZF3 is targeted in the processing bodies (PBs) and stress granules (SGs). Heterologous expression of *HuTZF3* could improve both salt and heat tolerance in *Arabidopsis*, reduce oxidative stress, and improve the activity of catalase and peroxidase. Therefore, HuTZF3 may be involved in post-transcriptional regulation via localizing to PBs and SGs, contributing to both salt and heat tolerance in pitaya.

## 1. Introduction

Soil salinity and increasing temperature caused by human activities, are two major environmental factors affecting plant growth and production [1,2]. To face salt and heat stress, plants initiate a series of physiological and biochemical reactions, to reduce damage and maintain adequate growth [3]. Pitaya (dragon fruit) is one of the tropical fruits belonging to the genus *Hylocereus*, in the Cactaceae family, with high commercial and medical value [4]. Pitaya is cultivated in a broad ecological range, due to its tolerance to drought, heat, and poor soil [5,6]. Pitaya performs crassulacean acid metabolism (CAM)-type photosynthesis [7], and its stems have spines and no leaves. Pitaya, like other CAM plants, has high water-use efficiency, due to the unique stomatal regulation pattern. The stomata of CAM plants open for CO_2_ uptake and fixing at night with lower temperature and higher humidity, and close for reducing water loss in the daytime when the stored CO_2_ is re-fixed by Rubisco for sugar synthesis [8,9,10]. So, CAM plants can endure various stresses including drought, salinity, and heat, and successfully survival in various ecological habitats, from deserts to forests [11].

There are many studies at transcriptomic and proteomic levels exploring the molecular mechanism in pitaya response to abiotic stresses including drought, salt, cold, and heat [5,6,12,13]. The pitaya catalase gene *HuCAT3*, is induced by H_2_O_2_, cold, drought, and salt stress, and plays an essential role in pitaya’s abiotic stress tolerance [14]. *HuERF1* is induced by salt stress and participates in the ethylene-mediated salt tolerance of pitaya [15]. The pitaya miR396b is involved in response to drought, cold, heat, salt, and abscisic acid (ABA), by regulating its target gene *HpGRF6* [16]. *HuPR-1*, a pathogenesis-related protein 1, is induced by heat stress, and overexpressing *HuPR-1* improved *Arabidopsis* heat tolerance [6]. The genome of pitaya has been sequenced, it is diploid (2n = 22 chromosomes) [17]. However, little is known about the genes responding to both salt and heat stress in pitaya.

The zinc finger proteins are a large class of transcription factors containing zinc finger domains, and regulate gene expression at the transcriptional and post-transcriptional levels by interacting with DNA, RNA, and proteins, and then play important roles in plant growth and development, and stress response [18]. The number and arrangement of Cys and His residues in the zinc finger proteins, can be divided into nine categories, including C2H2, C2HC, C2HC5, CCCH, C3HC4, C4, C4HC3, C6, and C8 [19,20]. Among them, the CCCH-type zinc finger proteins usually contain 1–6 copies of CCCH-type motifs of C-X_4-15_-C-X_4-6_-C-X_3-4_-H (X represents other amino acids). Tandem CCCH zinc finger (TZF) protein contains two CCCH motifs in tandem. The plant TZF protein contains a plant-unique arginine-rich (RR) motif (C-X_7–8_-C-X_5_-C-X_3_) in the front of the TZF motif [21,22]. Plant TZF proteins are further divided into two groups: the RR-TZF group, containing the arginine-rich (RR) and TZF domains, and the ANK-RR-TZF group, containing the Ankyrin repeat (ANK) and RR-TZF domains [21]. The ANK repeat motif is known as a protein–protein interaction motif and plays a role in plant growth and development [23]. Both RR and TZF domains can bind RNA, leading to the critical role of TZFs in regulating RNA metabolism [24].

Most TZFs are RNA binding proteins involved in RNA regulation, and localized to processing bodies (PBs) and stress granules (SGs). PBs and SGs are two cytoplasmic mRNP granules containing messenger ribonucleoprotein, and play important roles in post-transcriptional and translational levels. PBs are involved in mRNA decay, while SGs hinder translation initiation, but allow elongation [25,26]. AtTZF1/AtC3H23 can regulate gene expression at the RNA level by binding RNA, and participate in flowering, cold, and drought tolerance [27,28]. AtTZF2/AtC3H20 and AtTZF3/AtC3H49 have RNase activity in vitro, to promote the degradation of target mRNAs, and participate in RNA metabolism [29]. OsTZF1 affects RNA stability via binding mRNA containing the AU-rich motif in 3‘UTR, to delay senescence and improve plant stress tolerance [30]. OsTZF7 is localized to PBs and SGs, and downregulates target gene expression by binding the ARE motif in the mRNA 3′UTR region, to enhance drought tolerance in rice [31].

In this study, we analyzed the response of *HuTZF3* and the CCCH genes family of pitaya, to salt and heat stress. A total of 81 *CCCH* genes were identified from the pitaya genome, and the expression of nine of the genes responds to both salt and heat stress. Heterologous expression of *HuTZF3* could improve the tolerance of *Arabidopsis* to both salt and heat stress, and HuTZF3 is localized in PBs and SGs. Therefore, we deduced that HuTZF3 might affect RNA stability, to mediate the salt and heat tolerance.

## 2. Results

### 2.1. Identification of the CCCH-Type Zinc Finger Protein Genes from Pitaya

**Figure 1:**
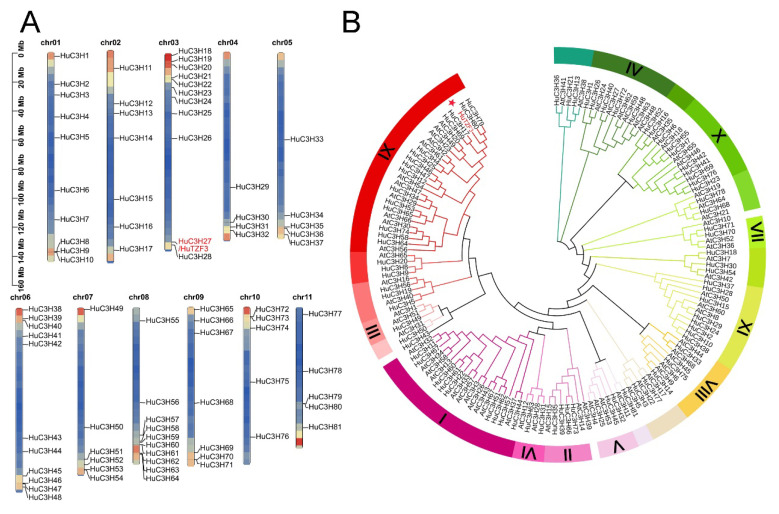
Systematic identification of the CCCH-type zinc finger protein genes from pitaya. (**A**) Chromosomal locations of pitaya *CCCH* genes. The chromosomal locations of *HuCCCH* genes were mapped by TBtools [32]. Names of the *HuCCCH* genes are shown on the right of the chromosomes. The scale of the genome size is shown on the left. (**B**) Phylogenetic tree of the CCCH proteins from pitaya and *Arabidopsis*. The 81 HuCCCH proteins from pitaya and the 68 AtCCCH proteins from *Arabidopsis* were used to construct the phylogenetic tree by MAG11 and visualized by iTOL. The red star indicates HuTZF3.

To identify the CCCH-type zinc finger protein genes from pitaya, the Pitaya Genomic Database was searched, and the CCCH zinc finger domain was confirmed in SMART, NCBI conserved domain search tools, and the Pfam database. A total of 81 CCCH-type zinc finger protein genes (*HuCCCHs*) were obtained (Figure 1 and Supplementary Table S1), and they were randomly distributed on the 11 chromosomes of pitaya (Figure 1A). These *HuCCCHs* were named from *HuCCCH1* to *HuCCCH81*, based on their chromosomal location, and their information is listed in Supplementary Table S1. In order to investigate their phylogenetic relationship, we constructed a phylogenetic tree with the CCCH-type zinc finger proteins from pitaya and *Arabidopsis* (Figure 1B). The pitaya HuCCCH proteins could be divided into 11 subfamilies, like those of *Arabidopsis*.

Since *HuTZF3* belongs to subfamily IX of the *HuCCCH* family (Figure 1B), we analyzed the conserved motif, conserved domain, *cis*-elements in promoter, and gene structure of the 16 TZF-type zinc finger protein genes in the subfamily IX (Supplementary Figure S1 and Table S3). The promoter *cis*-element analysis showed that there are many *cis*-elements in the promoter of *HuTZFs*, such as plant growth and development, hormone, and abiotic stress-related *cis*-elements (Supplementary Figure S1B). According to the phylogenetic tree, conserved motif, and conserved domain, the 16 pitaya TZF proteins were divided into two groups: the RR-TZF group, including *HuTZF1-10*, and the ANK-RR-TZF group, including *HuTZF11-16*, similar to that of *Arabidopsis* (Supplementary Figure S2). All HuTZFs of the subfamily IX have the RR-TZF domain, that the RR (arginine-rich) domain localized in front of the TZF domain (Supplementary Figure S2A). *HuTZF11-16* contains the ANK (Ankyrin repeat) domain and the RR-TZF domain belongs to the ANK-RR-TZF group (Supplementary Figure S2B).

### 2.2. Identification of HuCCCHs Response to Heat and Salt Stress

**Figure 2:**
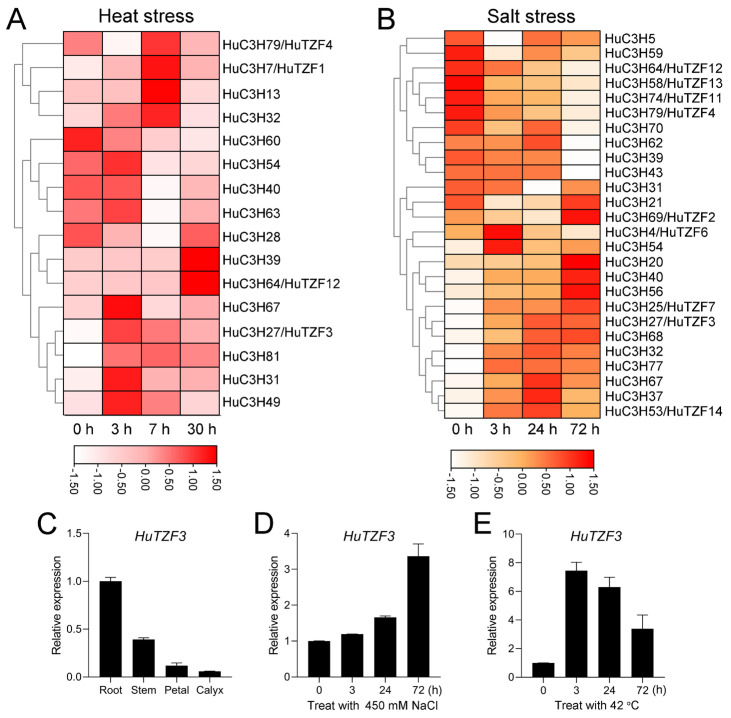
Expression response of pitaya *CCCH* genes to salt and heat stress. (**A**,**B**) Heatmap of *HuCCCHs* response to heat (**A**) and salt (**B**) stress. The transcriptomic data cited are from Nong et al. (2019) [5] and Jiao et al. (2021) [6]. The three-month-old seedlings grown in a greenhouse, were treated with 450 mM NaCl, or at 42 °C, for different times, then the samples were collected for RNA-seq. (**C**–**E**) Expression pattern of *HuTZF3* in different tissues of pitaya (**C**), and in pitaya seedlings under salt (**D**) and heat (**E**) treatment. Three-month-old pitaya seedlings were treated with 450 mM NaCl (**D**) at 42 °C (**E**). *HuEF1-α* was used as the internal reference gene. Values represent means ± SD (*n* = 3 technical repetitions). All RT-qPCR analyses for gene expression were performed in three biological replicates, with similar results.

To screen which *HuCCCHs* respond to both heat and salt stress, we analyzed our previous transcriptomic data of pitaya seedlings under salt and heat treatment [5,6]. We found that sixteen of the *HuCCCHs* responded to heat treatment (Figure 2A), and 26 genes responded to salt treatment (Figure 2B). Among them, nine genes were induced by both salt and heat stress, and they are *HuTZFs3/4/12* and *C3H31/32/39/40/54/67*. To verify this result, the expression pattern of *HuTZF3* was analyzed in different tissues and response to salt and heat stress by RT-qPCR (Figure 2C–E). The results showed that expression of *HuTZF3* was detected in different tissues. *HuTZF3* had a high expression level in roots and stems, and a low expression level in petal and calyx (Figure 2C). The expression of *HuTZF3* gradually increased under salt stress (Figure 2D), and its expression was rapidly induced by heat stress and reached a peak at 3 h (Figure 2E), which confirmed the screening result above from the transcriptomic data [5,6].

### 2.3. Heterologous Expression of HuTZF3 Improved Salt and Heat Tolerance in Arabidopsis

**Figure 3:**
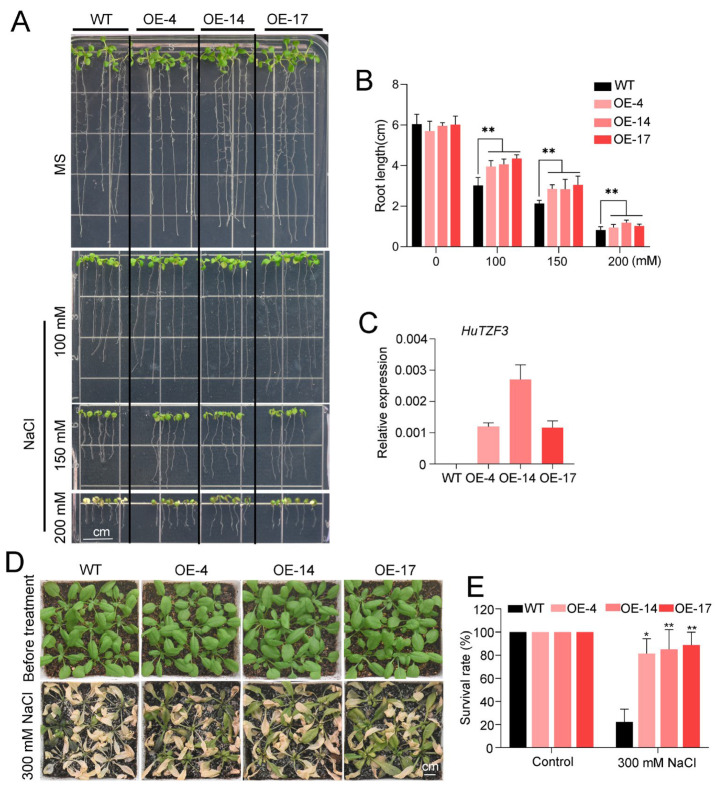
Heterologous expression of *HuTZF3* improved salt tolerance in *Arabidopsis.* (**A**,**B**) Phenotype of seedlings’ root growth (**A**) and statistical analysis of primary root length (**B**) on MS medium with NaCl. Five-day-old *Arabidopsis* seedlings were transferred to MS medium, containing 0, 100, 150, and 200 mM NaCl and cultured for 7 days. The scale bar is 1 cm. (**C**) The expression level of *HuTZF3* in the wild type (WT) and overexpressed *HuTZF3* transgenic *Arabidopsis* lines. *AtActin2* was used as the internal reference gene. (**D**,**E**) Four-week-old *Arabidopsis* of WT and overexpressing *HuTZF3* lines were subjected to 300 mM NaCl treatment (**D**), and their survival rate (**E**). The scale bar is 1 cm. The experiments were performed three times with similar results. Values are means ± SD (*n* = 30 plants). Asterisks show the values that are significant compared to WT. * *p* < 0.05, ** *p* < 0.01 according to the Student’s *t*-test.

To study whether *HuTZF3* affects the salt and heat tolerance of plants, we heterologously expressed *HuTZF3* in *Arabidopsis* with *CaMV35S* promoter (Figure 3). Three independent homozygous *HuTZF3* overexpressing lines (OE-4/14/17), showed high expression of *HuTZF3* (Figure 3C), and were selected for further study. First, the salt tolerance of these transgenic *Arabidopsis* was tested under different concentrations of NaCl, using the seedlings. When the five-day-old seedlings were transferred to MS medium, containing 0, 100, 150, and 200 mM NaCl, and then cultured for 7 days, the root length of the OE lines was significantly longer than that of the wild type (WT); however, there was no significant difference between the WT and OE lines when they were grown on MS without NaCl (Figure 3A,B). When the four-week-old *Arabidopsis* of the WT and OE lines were grown in soil and then subjected to 300 mM NaCl, the OE lines also showed a higher survival rate to salt stress than the WT (Figure 3D,E).

**Figure 4:**
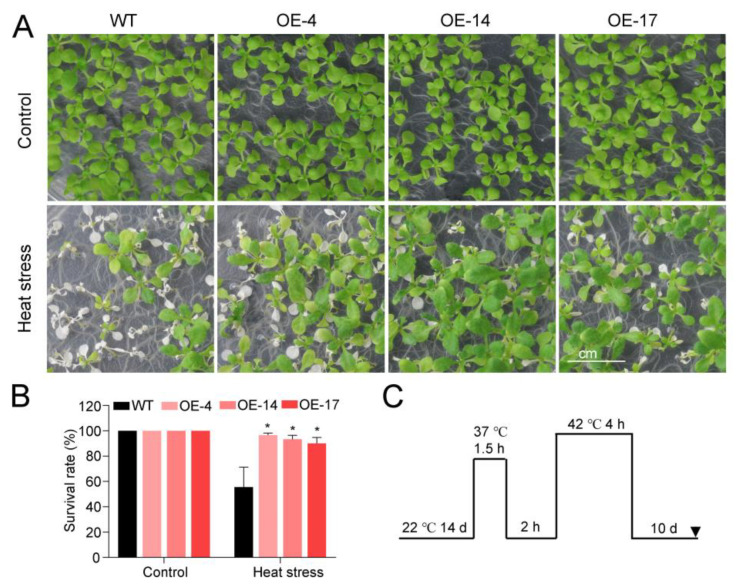
Heterologous expression of *HuTZF3* enhanced tolerance to heat stress in *Arabidopsis.* (**A**,**B**) Two-week-old *Arabidopsis* WT and overexpressing *HuTZF3* plants were exposed to heat treatment (**A**), and their survival rate (**B**) after recovering. The experiments were repeated three times with similar results. Values are means ± SD (*n* = 40 plants). Asterisks show the values that are significant compared to WT. * *p* < 0.05 according to the Student’s *t*-test. The scale bar is 1 cm. (**C**) Schematic diagram of heat treatment conditions.

The heat tolerance of the transgenic *Arabidopsis* was also tested (Figure 4). When the two-week-old seedlings were exposed to heat (42 °C) and recovered under normal temperature (22 °C) (Figure 4C), the survival rate of the seedlings in the OE lines was higher than that of the WT (Figure 4A,B). The OE lines had a more than 90% survival rate, whereas WT was only about 44%. These results indicate that heterologous overexpression of *HuTZF3* in *Arabidopsis* could improve its salt and heat tolerance.

### 2.4. Heterologous Expression of HuTZF3 Repressed Burst of Oxidative Stress in Arabidopsis

**Figure 5:**
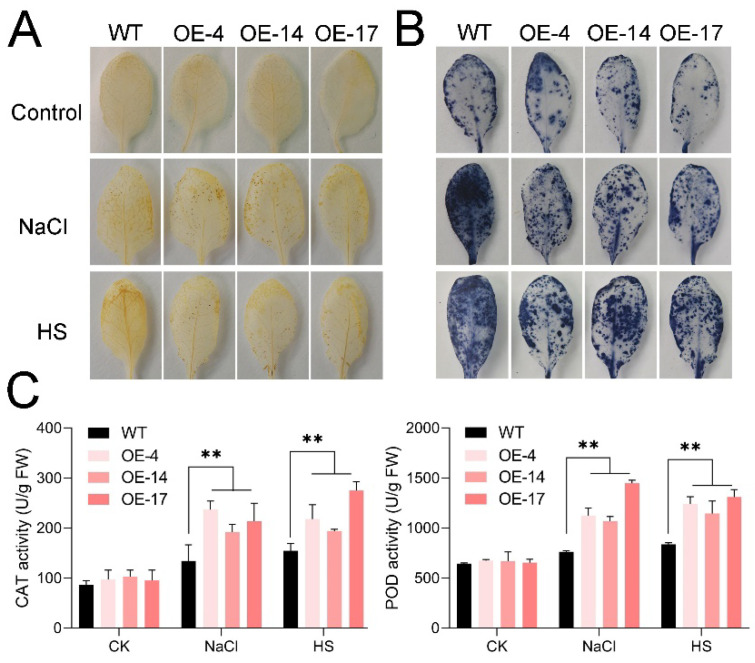
Oxidative stress analysis of the transgenic *Arabidopsis* with *HuTZF3* overexpression. (**A**,**B**) Detection of H_2_O_2_ and O_2_^−^ by DAB (**A**) and NBT (**B**) staining. (**C**) The activity of CAT and POD under salt and heat treatments. Four-week-old *Arabidopsis* WT and overexpression *HuTZF3* plants were exposed to 300 mM NaCl for 4 h, and 42 °C for 4 h. Three replicates of the experiments were performed. Values are means ± SD (*n* = 3). Asterisks show the values that are significant compared to WT. ** *p* < 0.01 according to the Student’s *t*-test.

To analyze the accumulation of ROS in WT and the *HuTZF3* overexpression lines, under salt and heat stress, the leaves were stained with DAB and NBT, to detect the production of H_2_O_2_ and O_2_^−^. The results showed that the content of H_2_O_2_ and O_2_^−^ in the WT accumulated more than in the *HuTZF3* overexpression lines (Figure 5A,B); however, there was no significant difference between the transgenic lines and WT plants in the absence of stresses (Figure 5A,B). The activities of CAT and POD in the *HuTZF3* OE lines were higher than in the WT, under salt and heat stress (Figure 5C). These results demonstrate that overexpression of *HuTZF3* can improve the ROS scavenging ability of *Arabidopsis*, to reduce oxidative stress under salt and heat treatments.

### 2.5. HuTZF3 Is Co-Localized with PBs and SGs Markers in Arabidopsis

**Figure 6:**
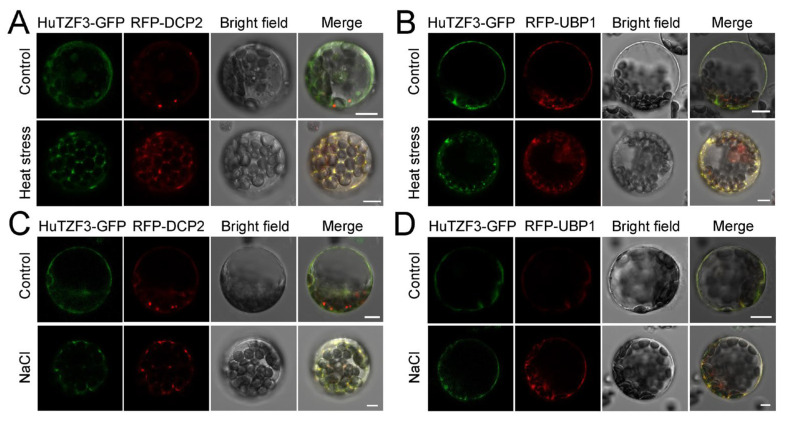
HuTZF3 co-localized with RNA processing bodies (PBs) and stress granules (SGs) marker proteins under heat and salt stress. (**A**,**B**) The *HuTZF3-GFP* was co-transformed with the PB marker *RFP-DCP2* (**A**) and the SG marker *RFP-UBP1* (**B**) into *Arabidopsis* protoplasts, and then cultured under control conditions (22 °C), or heat stress, at 39 °C for 30 min, before observation. (**C**,**D**) The *HuTZF3-GFP* was co-transformed with the PB marker *RFP-DCP2* (**C**) and the SG marker *RFP-UBP1* (**D**) into *Arabidopsis* protoplasts, and then cultured under control conditions, or salt stress with 150 mM NaCl for 1 h, before observation. Scale bar = 10 μm.

Since most TZFs are RNA-binding proteins involved in RNA regulation, and localized to PBs and SGs, the mRNA degradation factor DCP2 has decapping activity and is involved in PBs assembly, and the RNA-binding protein UBP1B, is required for SG formation. Therefore, DCP2 and UBP1B are known as PB marker and SG marker, respectively [25]. To explore whether *HuTZF3* is localized to PBs and SGs, subcellular localization of *HuTZF3* was performed. Protoplasts isolated from *Arabidopsis* leaves were co-transformed with *HuTZF3-GFP* and PBs marker *RFP-DCP2* or SGs marker *RFP-UBP1* (Figure 6). The HuTZF3-GFP was diffusely localized in the cytoplasm under the control condition. However, after heat (Figure 6A,B) and salt treatments (Figure 6C,D), HuTZF3-GFP was redistributed to cytoplasmic foci, to co-localize with the PBs and SGs markers. Although SG cytoplasmic foci were not evident under normal conditions, SG foci appeared in the cytoplasm under heat and salt stress (Figure 6B,D). These results showed that HuTZF3 is mainly localized in the cytoplasm under normal conditions and assembled into cytoplasmic foci to co-localize with PBs and SGs during the salt and heat treatment, indicating that HuTZF3 may participate in the RNA processing.

## 3. Discussion

The CCCH-type zinc finger proteins have been confirmed to play important roles in plant growth, development, and stress adaption. Here, we identified a total of 81 *CCCH* genes from pitaya, which were divided into 11 subfamilies based on the classification in *Arabidopsis* [21], and nine of them responded to both heat and salt stress (Figure 1 and Figure 2), indicating that the nine CCCH genes may play roles in the tolerance of pitaya to both salt and heat stress. HuTZF3 may affect RNA processing to play a role in salt and heat tolerance of pitaya, since it is localized in the PBs and SGs (Figure 6). The IX subfamily of the *HuCCCH* family, contained 16 HuTZFs, which are characterized by two CCCH zinc finger motifs arranged in tandem (Figure S2). Based on the presence of the Ankyrin repeat (ANK) domain, the *16 HuTZFs* were further divided into two groups: RR-TZF group and ANK-RR-TZF group, which is consistent with phylogenetic analysis of the CCCH genes in *Arabidopsis*, rice, and maize [21,33]. Promoter *cis*-element analysis showed that *HuTZFs* are involved in plant growth and development, and respond to multiple hormones and environmental stress (Figure S1), possibly like other plants [34,35,36]. Analysis of the transcriptomic data showed that the expression of nine genes could be regulated by both salt and heat stress (Figure 2A), implying that they may play roles in pitaya’s tolerance to heat and salt stress.

Heterologous expression of *HuTZF3*, confirmed that some pitaya TZFs play a role in tolerance to salt and heat stress (Figure 3 and Figure 4). *HuTZF3* is expressed in different organs of pitaya, with a high level in the roots and stems, and its expression in pitaya seedlings is induced by both salt and heat stress (Figure 2). This result implies that *HuTZF3* may contribute to pitaya tolerance to salt and heat stress, like *AtTZF1-3* [27,29] and rice *OsTZF1*,*5*,*8* [30,37,38]. Salinity and heat stress induced excessive ROS production and ultimately lead to oxidative stress. DAB and DBT staining revealed more ROS accumulation in the WT *Arabidopsis* than the *HuTZF3* OE lines under salt and heat stress (Figure 5A,B), the activities of CAT and POD in the *HuTZF3* OE lines were higher than in WT *Arabidopsis*, under salt and heat stress (Figure 5C). These results indicate that *HuTZF3* could improve tolerance to oxidative stress, to confer salt and heat stress, like rice *OsTZF1* [30].

In eukaryotes, cellular mRNAs are coated with proteins forming messenger ribonucleoprotein (mRNP) complexes [39]. The mRNP complex involves post-transcriptional regulation via controlling mRNA’s whole life cycle, from pre-mRNA processing to mRNA transport, localization, stability, and translation [40]. Many inactive translation mRNAs often assemble with proteins to format cytoplasmic mRNP granules. Processing bodies (PBs) and stress granules (SGs) are two well-characterized mRNP granules, that are widely appear during various stresses [41,42]. PBs have a major involvement in mRNA decay and translation repression, SGs also contain many stalled preinitiation complexes [43]. Our results indicated that HuTZF3 can localize to PBs and SGs under salt and heat stress (Figure 6). It is known that PB- and SG-localized OsTZF1 and OsTZF7 affect RNA stability, via binding mRNA, to delay senescence and improve plant stress tolerance [30,31]. The salt and heat stress could promote accumulation of HuTZF3 in PBs and SGs (Figure 6), this suggests that HuTZF3 might also be involved in the regulation of targeting RNA metabolism in post-transcriptional and translational levels, to confer salt and heat stress in pitaya. 

## 4. Materials and Methods

### 4.1. Plant and Growth Conditions

Pitaya (*Hylocereus polyrhizus*) and *Arabidopsis* were used as the plant materials in this experiment. The pitaya seeds were germinated and cultivated in the plant growth room (25 °C, 16 h/8 h light/dark photoperiod). *Arabidopsis* was cultivated in the plant growth room (22 °C, 16 h/8 h light/dark photoperiod).

### 4.2. Abiotic Stress Treatment

For pitaya stress treatment, pitaya seeds were germinated in the soil and the seedlings were grown in plates filled with nutrient soil at 25 °C, the pitaya plants were subjected to salt or heat stress. For salt on primary root growth assay, *Arabidopsis* seeds were germinated vertically on MS medium for 5 days, then seedlings of similar root length were chosen to transfer to MS medium containing 0, 100, 150, and 200 mM NaCl, and vertically cultured for 7 days. For *Arabidopsis* stress treatment, *Arabidopsis* seedlings were transferred and grown in nutrient soil, after germination on MS medium, for 7 days, then four-week-old plants were watered with a solution with 300 mM NaCl for salt stress, or exposed to 42 °C in an incubator for heat stress.

### 4.3. Sequence Analysis of HuTZF Genes

The pitaya CCCH-type zinc finger proteins were obtained from the Pitaya Genomic Database (http://www.pitayagenomic.com/index.php) (accessed on 9 August 2022) [17,44]. Further, the sequences were confirmed by SMART (https://smart.embl.de/)(accessed on 10 August 2022) NCBI conserved domain search tools (https://www.ncbi.nlm.nih.gov/Structure/cdd/wrpsb.cgi) (accessed on 10 August 2022), and Pfam (https://pfam.xfam.org/)(accessed on 10 August 2022). The sequence of AtCCCH proteins were referred to Wang et al. [21] and downloaded from TAIR (TAIR—home page (arabidopsis.org)) (accessed on 9 August 2022). The phylogenetic tree was constructed by the neighbor-joining (NJ) method, with 1000 bootstraps, in MAGE11, then visualized and optimized by iTOL (https://itol.embl.de/)( accessed on 30 August 2022). The multiple sequence alignment of the conserved domains was analyzed by GENEDOC.

### 4.4. RNA Isolation and RT-qPCR Analysis

Total RNA was extracted from pitaya and *Arabidopsis* using the Eastep Super Total RNA Extraction Kit (Promega, Beijing, China). First-strand cDNA was synthesized using GoScript^TM^ Reverse Transcription Mix (Promega, Beijing, China). RT-qPCR reactions were performed with MonAmp^TM^ ChemoHS qPCR Mix (Monad, Wuhan, China), by Roche Light Cycler 480 Real-time PCR System (Roche, Basel, Switzerland). *Arabidopsis Actin2* and pitaya *HuEF1-α* were used as internal reference genes [45]. The primers of RT-qPCR are listed in Supplementary Table S2.

### 4.5. Vector Construction and Genetic Transformation

The full-length *HuTZF3* cDNA was cloned into the pCAMBIA1302 vector driven by *CaMV35S*, to construct the *HuTZF3* overexpression vector. *Arabidopsis* transgenic plants were produced by the Agrobacterium-mediated floral dipping method [46]. Positive transgenic plants were screened on MS medium by kanamycin and confirmed by PCR, until transgenic homozygous lines were obtained.

### 4.6. Histochemical and Physiological Analysis of Oxidative Stress

After the heat and salt treatment for 4 h, leaves were submerged in 1 mg/mL DAB or 1 mg/mL NBT solution within 10 h, for in situ detection of the superoxide (O_2_^−^) anion and hydrogen peroxide (H_2_O_2_), and washing in 95% ethanol [47]. Peroxidase (POD) and catalase (CAT) activities were measured using POD and CAT Assay Kits (Nanjing Jiancheng, Nanjing, China), according to the manufacturer’s instructions.

### 4.7. Subcellular Localization of HuTZF3

The coding sequences of *HuTZF3* were inserted into the pUC/GFP vector, to construct a fusion plasmid (*HuTZF3-GFP*). The RFP fusion plasmids were constructed by adding the coding sequence of *DCP2* and *UBP1* to pBI221/RFP, which were as described in [48]. *Arabidopsis* mesophyll protoplasts were isolated from four-week-old *Arabidopsis* leaves and transformed by PEG-CaCl_2_-mediated transfection. After incubating the transformed protoplasts at room temperature for 12–16 h in darkness, the protoplasts were then subjected to 39 °C for heat stress, or 150 mM NaCl for salt stress. Protoplasts were observed by using confocal laser microscopy Leica SP8 STED 3X (Leica Microsystems, Mannheim, Germany).

## 5. Conclusions

To comprehensively examine the CCCH gene family in pitaya, a genome-wide investigation identified 81 *HuCCCHs*, which were classified into 11 subfamilies. Analysis of the transcriptomic data found that 9 *HuCCCHs*, including *HuTZF3,* responded to both salt and heat stress. *HuTZF3* is constitutively expressed in different organs of pitaya, with a high level in the roots and stems. RT-qPCR confirmed that expression of *HuTZF3* is induced by both salt and heat stress. Overexpression of *HuTZF3* improved *Arabidopsis* tolerance to salt and heat stress. Co-localization of *HuTZF3* with PBs and SGs was observed under salt and heat stress. Our findings suggest that *HuTZF3* may participate in RNA metabolism to cope with the salt and heat stress of pitaya.

## Data Availability

The data that support the findings of this study are available in the supplementary material of this article.

## Supplementary Materials

The following supporting information can be downloaded at: https://www.mdpi.com/article/10.3390/ijms24076359/s1.
