# Supplementary material for: Genomic Identification of CCCH-Type Zinc Finger Protein Genes Reveals the Role of HuTZF3 in Tolerance of Heat and Salt Stress of Pitaya (Hylocereus polyrhizus)
Source: Int J Mol Sci. 2023 Mar 28;24(7):6359. doi: 10.3390/ijms24076359 (PMC10094633; doi:10.3390/ijms24076359)
Supplement: Supplementary file 1 [file ijms-24-06359-s001.zip › Supplement Figure.pdf]

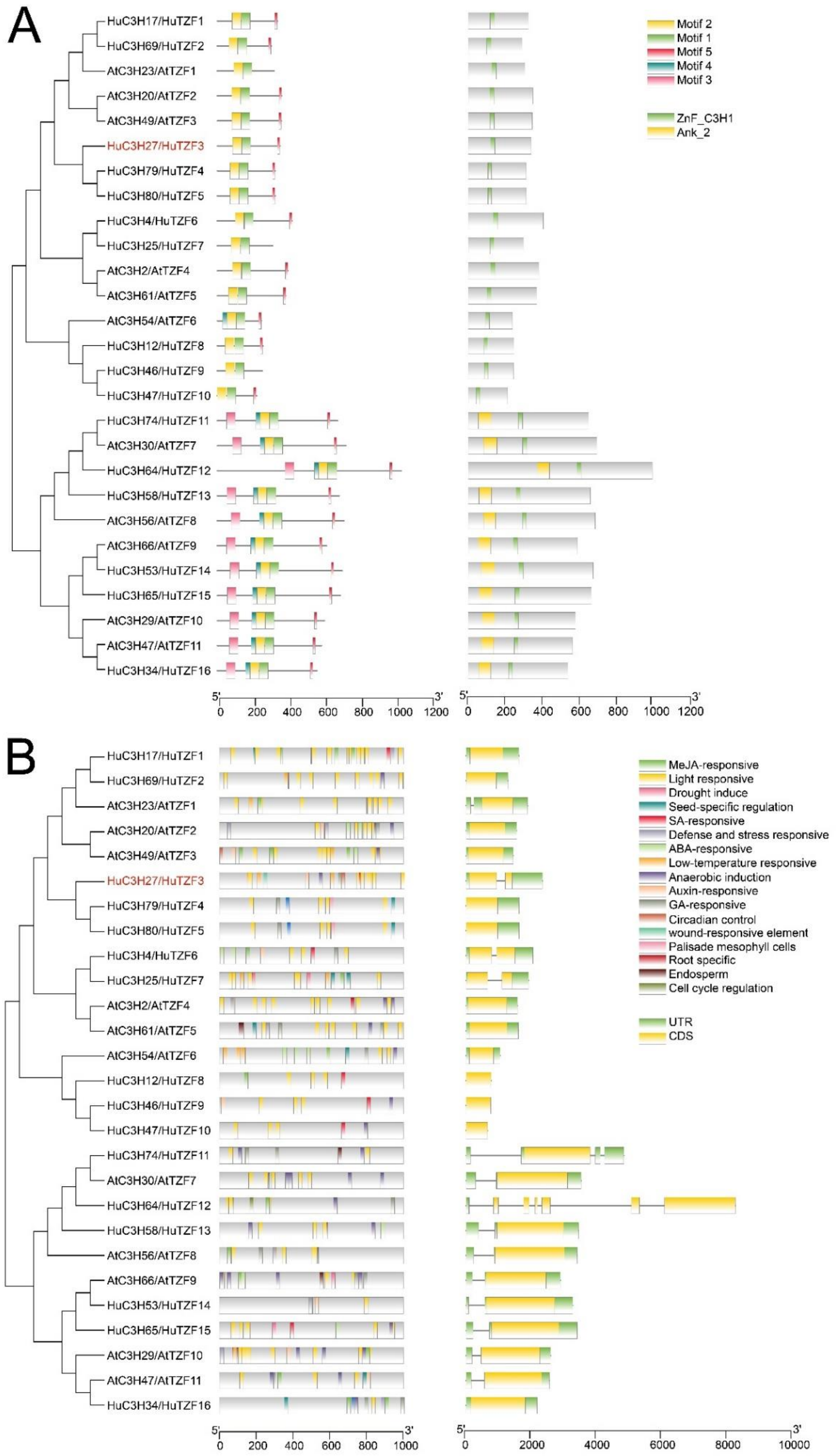

Supplementary Figure S1. Phylogenetic analysis, conserved motifs, conserved

domains, *cis*-acting regulatory elements in promoters, and gene structure of subfamily IX in the *CCCH* genes.**(A)** Phylogenetic tree was analyzed by MEGA11, conserved motif by MEME and conserved domain by NCBI conserved domains database (CDD). **(B)** *cis*-elements in promoter by Plant Care and gene structure by TBtools. Visualized by TBtools.

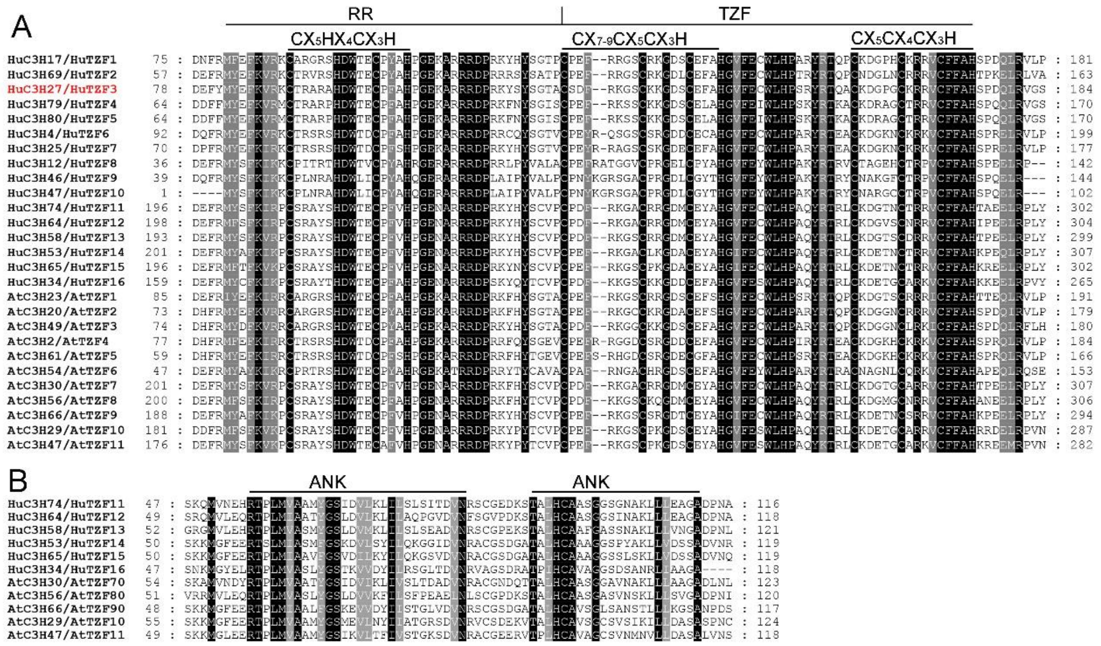

Supplementary Figure S2. Sequence analysis of subfamily IX in pitaya and *Arabidopsis*. Amino acid sequence alignment of the zinc finger motifs **(A)** and ankyrin repeat (ANK) domains **(B)** in the subfamily IX.
