# Supplementary material for: Genomic Identification of CCCH-Type Zinc Finger Protein Genes Reveals the Role of HuTZF3 in Tolerance of Heat and Salt Stress of Pitaya (Hylocereus polyrhizus)
Source: Int J Mol Sci. 2023 Mar 28;24(7):6359. doi: 10.3390/ijms24076359 (PMC10094633; doi:10.3390/ijms24076359)
Supplement: Supplementary file 1 [file ijms-24-06359-s001.zip › Supplement Table.pdf]

Supplementary Table S1. List of identified *HuCCCH* genes in pitaya along with their chromosomal locations and proteomic information

Gene name	Accession number	Chrom	Chr start	Chr end	Protein/AA	pI	MW(Da)
HuC3H1	HU01G00226.1	chr01	2782537	2787570	696	5.67	77095.87
HuC3H2	HU01G00905.1	chr01	22038524	22042315	319	7.19	50282.03
HuC3H3	HU01G01005.1	chr01	29390111	29399239	881	8.08	101955.37
HuC3H4	HU01G01125.1	chr01	44553152	44555195	417	6.73	45921.28
HuC3H5	HU01G01231.1	chr01	58827319	58864270	426	8.44	48226.21
HuC3H6	HU01G01390.1	chr01	95239847	95242439	563	5.94	63730.67
HuC3H7	HU01G01629.1	chr01	115446115	115449452	580	6.05	64701.97
HuC3H8	HU01G02324.1	chr01	137196357	137203757	1397	6.13	151482.42
HuC3H9	HU01G02326.1	chr01	137221973	137229370	1396	6.24	151511.49
HuC3H10	HU01G02363.1	chr01	137681275	137693558	903	5.88	96962.8
HuC3H11	HU02G00985.1	chr02	12433649	12444163	457	8.5	48343.21
HuC3H12	HU02G02007.1	chr02	36884092	36884853	254	8.35	28754.36
HuC3H13	HU02G02106.1	chr02	43435336	43457666	826	8.83	92438.58
HuC3H14	HU02G02269.1	chr02	60362370	60366431	738	5.51	81746.19
HuC3H15	HU02G02494.1	chr02	102551084	102554444	440	9.22	51005.03
HuC3H16	HU02G02669.1	chr02	121734570	121744060	360	8.69	39780.29
HuC3H17	HU02G02936.1	chr02	137672084	137673698	335	6.98	37616.11
HuC3H18	HU03G00163.1	chr03	1509465	1524207	1657	4.89	179676.37
HuC3H19	HU03G00599.1	chr03	5551758	5553940	259	9.15	28699.12
HuC3H20	HU03G00716.1	chr03	6770502	6783097	1024	9.38	110505.44
HuC3H21	HU03G01444.1	chr03	16767055	16775811	150	9.04	16591.74
HuC3H22	HU03G01511.1	chr03	17698176	17704081	456	8.51	49097.26

HuC3H23	HU03G01615.1	chr03	20388828	20393662	691	5.88	76411.24
HuC3H24	HU03G01805.1	chr03	30188119	30191737	324	9.1	37911.65
HuC3H25	HU03G01941.1	chr03	41390457	41392367	308	8.57	34082.34
HuC3H26	HU03G02096.1	chr03	58672951	58692103	851	5.33	93357.47
HuC3H27	HU03G02697.1	chr03	129790146	129792479	349	6.79	38127.4
HuC3H28	HU03G02771.1	chr03	131771850	131775956	523	8.61	59187.1
HuC3H29	HU04G01184.1	chr04	93322569	93329865	311	9.45	36188
HuC3H30	HU04G01477.1	chr04	117282428	117306249	1390	6.18	155093.29
HuC3H31	HU04G01583.1	chr04	120561665	120572864	346	6.38	37905
HuC3H32	HU04G01723.1	chr04	123044963	123049093	459	6.38	37905
HuC3H33	HU05G00908.1	chr05	60062037	60072211	2137	8.69	234990.16
HuC3H34	HU05G01448.1	chr05	112291442	112293611	554	8.45	60515.27
HuC3H35	HU05G01720.1	chr05	119995504	119996669	295	8.66	32825.72
HuC3H36	HU05G01794.1	chr05	120967284	120978221	948	7.1	102543.87
HuC3H37	HU05G02078.1	chr05	125253402	125257593	1245	5.42	131570.17
HuC3H38	HU06G00107.1	chr06	1077446	1088794	1779	6.08	193654.93
HuC3H39	HU06G00645.1	chr06	7229598	7231022	278	8.62	30810.38
HuC3H40	HU06G00733.1	chr06	8466308	8477608	219	8.62	30810.38
HuC3H41	HU06G01113.1	chr06	19349432	19355056	748	6.33	80783.2
HuC3H42	HU06G01201.1	chr06	24971234	24977197	748	6.33	80783.2
HuC3H43	HU06G01678.1	chr06	89559418	89578867	1787	6.33	80783.2
HuC3H44	HU06G01752.1	chr06	98703880	98738621	548	8.31	58986.17
HuC3H45	HU06G02044.1	chr06	115376484	115383257	489	7.91	54869.83
HuC3H46	HU06G02348.1	chr06	120439699	120440451	251	8.94	28205.89
HuC3H47	HU06G02369.1	chr06	121061664	121062317	218	9.15	24522.97

HuC3H48	HU06G02465.1	chr06	123164262	123171862	440	7.92	47779.15
HuC3H49	HU07G00096.1	chr07	1020304	1029731	331	6.62	37685.93
HuC3H50	HU07G01420.1	chr07	82417745	82449760	1019	6.16	115469.56
HuC3H51	HU07G01781.1	chr07	104265779	104270496	435	6.16	47125.81
HuC3H52	HU07G01923.1	chr07	108062108	108064435	428	9.57	49121.93
HuC3H53	HU07G02156.1	chr07	111445768	111449028	695	5.91	75130.21
HuC3H54	HU07G02173.1	chr07	111580379	111584103	378	6.92	44330.79
HuC3H55	HU08G00286.1	chr08	9331386	9336277	565	6.27	63747.02
HuC3H56	HU08G00718.1	chr08	65667001	65677438	385	8.11	41424.03
HuC3H57	HU08G01111.1	chr08	87952373	87959381	532	5.57	59519.87
HuC3H58	HU08G01200.1	chr08	92148249	92151683	679	6.29	73956.25
HuC3H59	HU08G01222.1	chr08	92763966	92767848	695	5.95	76319.08
HuC3H60	HU08G01335.1	chr08	94571394	94576990	421	8.61	46124.01
HuC3H61	HU08G01341.1	chr08	94610342	94615933	381	7.54	41692.73
HuC3H62	HU08G01831.1	chr08	100435137	100445435	470	5.58	52093.41
HuC3H63	HU08G01864.1	chr08	100973028	100977131	337	8	37476.92
HuC3H64	HU08G01870.1	chr08	101037964	101046195	1025	6.48	113134.21
HuC3H65	HU09G00164.1	chr09	1738493	1741888	685	5.98	74254.45
HuC3H66	HU09G00410.1	chr09	9585879	9587969	352	8.96	38762.8
HuC3H67	HU09G00531.1	chr09	18066434	18073740	470	8.37	49606.62
HuC3H68	HU09G00811.1	chr09	66115372	66123221	369	5.42	41890.88
HuC3H69	HU09G01465.1	chr09	100270044	100271321	300	8.03	33742.94
HuC3H70	HU09G01797.1	chr09	105100988	105105980	293	9.41	30369.39
HuC3H71	HU09G01798.1	chr09	105107351	105112336	295	9.43	30606.69
HuC3H72	HU10G00596.1	chr10	6677862	6681081	462	7.19	50282.03

HuC3H73	HU10G00654.1	chr10	7593435	7596632	358	7.65	39024.68
HuC3H74	HU10G00982.1	chr10	14636546	14641363	669	5.98	73108.37
HuC3H75	HU10G01534.1	chr10	51609624	51623264	462	6.52	50262.78
HuC3H76	HU10G01711.1	chr10	89627952	89632785	691	5.88	76411.24
HuC3H77	HU11G00058.1	chr11	4866361	4873603	519	5.34	57585.26
HuC3H78	HU11G00295.1	chr11	44042651	44070714	611	6.19	68037.18
HuC3H79	HU11G00518.1	chr11	66200743	66202361	322	8.23	35741.22
HuC3H80	HU11G00519.1	chr11	66400641	66402260	322	8.23	35741.22
HuC3H81	HU11G00912.1	chr11	82816066	82834765	692	6.04	75457.76

Supplementary Table S2. Primer sequence information

Primer name	Sequence(5' to 3')	Notes
HuTZF3-OE-F-BglII	TGACCATGGTAGATCTGATGATGATCGGAGAAACACAC	*HuTZF3*-OE vector
HuTZF3-OE-R-SpeI	CTTCTCCTTTACTAGTCTTGACGAGATCGGACACC	construction
HuTZF3-GFP-F-SpeI	CGGACTAGTATGATGATCGGAGAAACACAC	*HuTZF3-GFP* vector
HuTZF3-GFP-R-KpnI	CGGGGTACCCTTGACGAGATCGGACACC	construction
In-RFP-UBP1-F-XbaI	CTGTACAAGTCTAGAATGCAGAGGTTGAAGCAGCA	*RFP-UBP1* vector
In-RFP-UBP1-R-SacI	GGGAAATTCGAGCTCTTACTGGTAGTACATGAGCTGCT	construction
In-RFP-DCP2-F-XbaI	CTGTACAAGTCTAGAATGTCGGGCCTCCATCGA	*RFP-DCP2* vector
In-RFP-DCP2-R-SacI	GGGAAATTCGAGCTCTCAAGCTGAATTACCAGA	construction
HuTZF3-qRT-F	CTGTTTCTTCGCCCACTCG	qRT-PCR primers of
HuTZF3-qRT-R	TCGGCATGGATTTCACCTT	*HuTZF3*
HuEF1-a-qRT-F	CTGGACAAATCGGAAACGGC	qRT-PCR primers of
HuEF1-a-qRT-R	ATGTCCCTCACAGCAAAACGA	*HuEF1-a*
AtACT2-qRT-F	ATTACCCGATGGGCAAGTCA	qRT-PCR primers of
AtACT2-qRT-R	TGCTCATACGGTCAGCGATAC	*AtACT2*

Supplementary Table S3. The motifs and motifs logo of HuTZF were predicted by MEME search

Name	Consensus sequence	Length (aa)	E-value
Motif 1	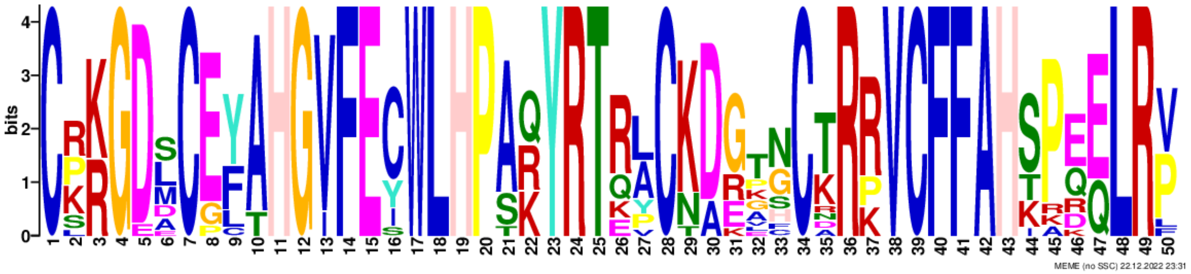	50	3.1e-582
Motif 2	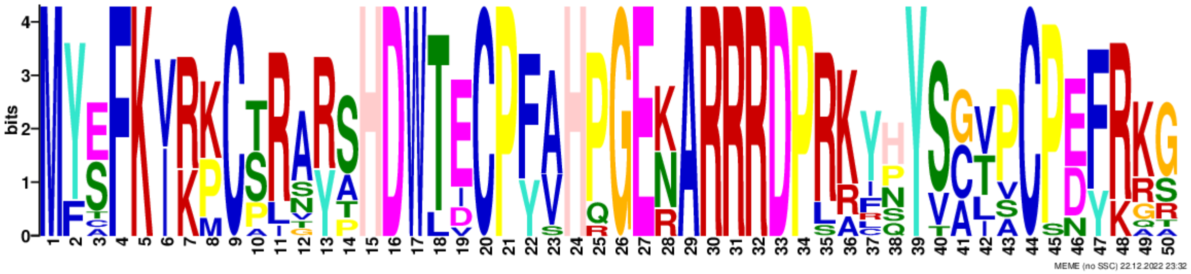	50	1.6e-555
Motif 3	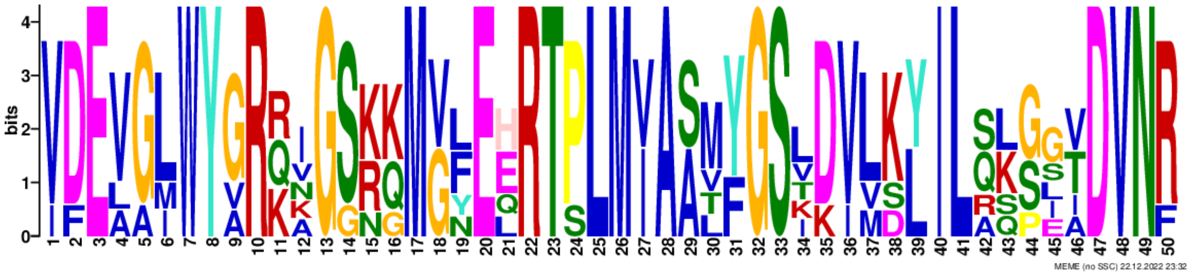	50	2.00E-110

Motif 4	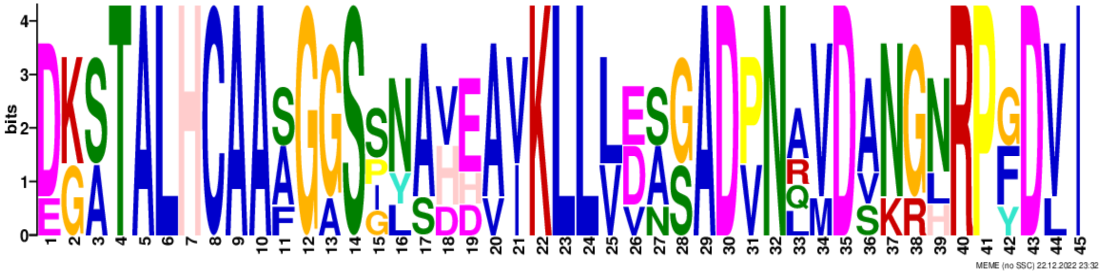	45	2.20E-65
Motif 5	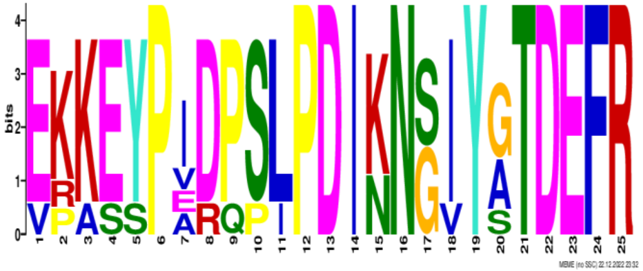	25	2.80E-64
